# Caregiver psychological burden of RSV Hospitalization of children 2 years of age and under

**DOI:** 10.1371/journal.pone.0334405

**Published:** 2025-10-30

**Authors:** Lazarus Adua, Krow Ampofo, Evan Heller, Per Gesteland, Abbey Loveridge, Katherine Werdan, Kaleb Miller, Alex Platt-Koch, Madelyn Ruggieri, Lyn Finelli, Yoonyoung Choi

**Affiliations:** 1 Department of Sociology and Criminology, University of Utah, Salt Lake City, Utah, United States of America; 2 Department of Pediatrics, University of Utah, Salt Lake City, Utah, United States of America; 3 Michigan State University, College of Osteopathic Medicine, East Lansing, Michigan, United States of America; 4 Outcomes Research, Merck & Co., Inc., Rahway, New Jersey, United States of America; Ain Shams University Faculty of Nursing, EGYPT

## Abstract

**Objectives:**

Caregiver psychological burden has been reported among premature children hospitalized with respiratory syncytial virus (RSV) infection. This study addresses two objectives: 1) assessing the psychological burdens of stress and anxiety among caregivers of otherwise healthy children 2 years of age and under hospitalized with RSV lower respiratory tract infection (LRTI); and 2) analyzing sociodemographic and clinical factors related to these psychological burdens.

**Study Design:**

We prospectively recruited caregivers of children ≤2 years of age hospitalized with RSV LRTI at Primary Children’s and Riverton Hospitals, Salt Lake City, Utah, during the 2019–2022 RSV seasons. Data came from a survey that included the Parental Stress Scale (PSS) and the State-Trait Anxiety Inventory for Adults (STAIAD) and patients’ medical records. Relationships between sociodemographic and clinical factors and caregiver stress and anxiety were assessed using linear regression models.

**Results:**

In all, 146 caregivers completed the survey in-hospital and 109 at 2 weeks post-discharge. Substantial levels of stress and anxiety were observed. Over 50% rated more than half of the PSS items as very or extremely stressful, and over 80% rated three specific items as extremely stressful: 87% felt helpless about how to help their child, 85% were concerned about their child’s unusual breathing, and 81% felt unable to protect their child from pain and painful procedures. On most STAIAD items, we observed an increase in positive outlook and a decrease in negative outlook items between hospitalization and two weeks post-discharge, with the greatest change seen in reduced worry about things that don’t matter. Caregiver’s increased stress and anxiety were significantly associated with Hispanic ethnicity, non-White race, lower household income, and more intensive respiratory support types. Household income was an important factor influencing the relationship between ethnicity and caregiver stress and anxiety.

**Conclusions:**

The hospitalization of children ≤2 years of age with RSV lower respiratory tract infection remains a major source of psychological burden on caregivers. The difference in burden by ethnicity, race and income identified highlights the importance of seeking equity in the distribution of RSV immunoprophylaxis and maternal vaccines for prevention.

## Introduction

Respiratory syncytial virus (RSV) is one of the most common causes of childhood respiratory infection worldwide [1–3]. By the second year of life, almost all children will experience RSV infection [4]. Primary RSV infection is almost always symptomatic in young children, with clinical manifestations ranging from mild upper respiratory-tract infection or otitis media to severe infection, like bronchiolitis and pneumonia [5,6]. In the U.S., over 2 million children ≤5 years of age are estimated to seek medical attention for RSV infection, with approximately 3% hospitalized, 25% managed in emergency departments, and 73% treated in outpatient pediatric practices [6].

The risk of RSV-associated hospitalization is highest in young children, with hospitalization rates of 48.9, 28.4, 13.4 and 5.0 per 1000 for children aged <3 months, 3–5 months, 6–11 months and 12–23 months respectively reported in the U.S. [6,7]. With RSV hospitalization, there is substantial hospital resource utilization, such as ICU space and the associated resources, steroids and antibiotics, and requirement of higher levels of respiratory support, when compared to other respiratory viral infections. Hospitalization also imposes burdens on parents and caregivers.

Thus far, a few studies of preterm infants in the neonatal ICU have evaluated caregiver stress and anxiety in children hospitalized with RSV infection in the U.S. [1,8]. Given that the majority of RSV-related hospitalizations, however, occur among otherwise healthy infants and young children, further understanding of its psychological burdens in the broader population of caregivers is needed [9]. Most prior studies examining stress and anxiety related to hospitalization of children with RSV infections have focused on children with chronic or other serious health conditions, which makes it necessary to consider the nature of these psychological burdens on caregivers of hospitalized children who are otherwise healthy. Furthermore, long-acting RSV monoclonal antibodies and maternal vaccines for prevention of RSV in infants will become widely available, [10,11] and comprehensive data on family impacts and the roles of race and ethnicity will be useful for healthcare providers.

We first evaluate levels of stress and anxiety among caregivers of otherwise healthy children ≤2 years of age hospitalized with laboratory-confirmed RSV infection with a physician-diagnosis of lower respiratory tract infection (LRTI). Second, we analyze sociodemographic and clinical factors related to these psychological burdens. Although caregivers experience the same event—a child’s RSV LRTI-related hospitalization—we hypothesize that their stress and anxiety levels will vary by sociodemographic and clinical characteristics. This hypothesis is informed by the *stress process model*, which among other propositions, argues that triggering events often worsen existing social strains or create new ones that vary across individuals and populations [12–14].

### The stress process model and psychological burdens

The stress process model proposes three conceptual domains for analyzing stress and anxiety: 1) source of stress or anxiety, which includes a precipitating event and the strains it causes or exacerbates, 2) stress mediators (social support and coping mechanisms), which can mitigate or exacerbate the situation; and 3) stress and anxiety manifestation, which entails how stress is empirically observed.[12–14]. The first domain applies to all caregivers in nearly the same manner, given that they all experience the hospitalization of child with RSV LRTI. The second domain applies to caregivers in a variegated manner, since they possess different types and levels of social support and coping mechanisms. This domain accounts for why caregivers of varying characteristics—ethnicity, race, income, and severity of illness of child hospitalized—may experience different levels of stress and anxiety. The third domain of the model is the manifestation of stress and anxiety, which is exemplified by what caregivers report. Thus, although caregivers experience the same precipitating event—RSV-related hospitalization of a child—we anticipate based on this model that the resulting stress and anxiety will vary along sociodemographic characteristics of the caregivers as well as clinical conditions of the hospitalized child. This is because the precipitating event likely will create new strains or intensify pre-existing ones related to caregivers’ different social contexts and the coping mechanisms they possess [15].

## Methods

### Research design and data

Caregivers of children ≤2 years of age hospitalized at Primary Children’s and Riverton Hospitals, Salt Lake City, Utah, with laboratory-confirmed RSV LRTI during the 2019−2022 RSV seasons were prospectively identified, recruited, and surveyed over two time periods. RSV LRTI diagnosis was identified using the following International Classification of Diseases, Tenth Revision (ICD-10) codes in the admission and discharge codes: bronchitis (J20.x), bronchiolitis (J21.x), and pneumonia (J09.X, J10, J12-18) of the lower respiratory tract. We adopted a prospective approach because it offered us the best opportunity to collect contemporaneous data on caregiver psychological burdens (stress and anxiety) related to the hospitalization of a child; hospitals do not routinely collect information on these variables to allow for a retrospective approach.

To qualify for inclusion in the study, caregivers must be of children hospitalized with RSV LRTI during the period specified, and they must be Utah residents. Hospitalization was defined as an inpatient stay in the short-stay unit, inpatient ward, or intensive care unit at the specified hospitals. In essence, the target population for the study was caregivers of all children ≤2 years of age hospitalized with RSV LRTI at the hospitals specified. All caregivers meeting this criterion were invited to participate in the study, giving each of them an equal chance of inclusion in the sample. In the end, about 35% consented and participated in the study. On two factors we can reasonably compare, our sample characteristics come close to Utah’s: in terms of race 81.5% of our respondents identified as White, compared with 82.4% for Utah as whole [16]; and in terms of income, the reported median income of our respondents was $89999.50, compared with $86833.00 for Utah [17]. The slight differences are consistent with our effective margin of error (see below).

Respiratory viral testing was conducted using nasopharyngeal swab specimens on two platforms: 1) the Cepheid GeneXpert Xpress System (Cepheid, Sunnyvale, CA); and 2) the BioFire® FilmArray Respiratory Panel 2.1 (Salt Lake City, UT). We only included caregivers of patients with positive test results for RSV infection during admission. To ensure that the inclusion criteria were closely adhered to, we cross-referenced admission and discharge diagnosis codes to confirm that RSV-positive patients had a physician-diagnosed RSV LRTI.

Prospective identification, recruitment, and surveying of study participants commenced on December 23, 2019 and ended on May 31, 2022. Written informed consent was obtained from all caregivers participating in the survey portion of the study. Consent was obtained from parents and/or guardians with legal authority of the child. All participants received a signed copy of the informed consent form; originals are maintained by the study team. For ethical and research integrity reasons, all participants were clearly advised that participation in the study was completely voluntary. We informed caregivers that they had the absolute right to refuse participation, and that they could terminate consent at any time during the data collection phase. The research was done under the auspices of the University of Utah Institutional Review Board (IRB), which ensured human subjects were adequately protected. It underwent a full IRB review, culminating in a grant of approval. No person was harmed or hurt in the research. The survey portion upon which this study is largely based did not rely on retrospective or pre-existing data. Supporting data—length of stay, highest level of care, level of oxygen support, health insurance status—were obtained prospectively after informed consent.

While at the hospital (during hospitalization of their child), each participating caregiver completed a questionnaire that was administered by an interviewer. Caregivers subsequently completed a self-administered follow-up survey (questionnaire) delivered electronically (email) approximately 2-weeks post-discharge. We estimated that a sample size of 50 would result in an expected margin of error (i.e., the probability that our sample results differ from the true population average) of 14.1%, while a sample size of 200 would reduce the margin of error to 7.1%. Therefore, we aimed to enroll 200 survey participants. Nevertheless, the sample we ended up with was adequate for the analysis conducted in the study [18,19].

The survey collected sociodemographic data, and also included two well-established psychometric instruments: the Parental Stress Scale (PSS) [20], which assesses caregivers’ levels of stress related to a child’s illness and hospitalization; and the State-Trait Anxiety Inventory for Adults (STAIAD) [21], to assess parental anxiety during hospitalization and 2 weeks after the child had been discharged from the hospital. We used these instruments because they have been applied widely in contexts similar to ours, proving effective in assessing stress and anxiety [22–24]. We also abstracted patients’ clinical data, shown in **Table 1**, from their medical records.

**Table 1:** Characteristics and demographics of enrolled caregivers and children hospitalized with laboratory-confirmed RSV infection (N = 146).

Characteristic	Percent Reporting/Mean
**Respondent (Caregiver)**	
Female (%)	115 (78.8)%
White (%)	119 (81.5%)
Hispanic (%)	27 (18.5%)
Age in years, mean (SE)	30.7 (0.35)
Mean annual household income ($)^a^	89417.30 (3391.66)
Health insurance status:	-
Private	130 (89%)
Medicaid	9 (6%)
Self-pay	7 (5%)
Out-of-pocket non-hospital costs occurred (%)	65.8%
**The Hospitalized Children (**≤2years)	
Age in months, mean (SE)	8.6 (SE)
Male (%)	80 (55%)
Mean length of hospital stay in days (SE)	3.5 (0.33)
ICU admission (%)	47 (32%)
Oxygen support level^b^	
None	13 (9%)
Standard oxygen therapy	65 (44.5%)
High-flow nasal cannula	25 (17.1%)
Non-invasive ventilation	33 (22.6%)
Invasive mechanical ventilation +/-ECMO	10 (6.9%)
Pre-existing chronic medical condition^c^	34 (23.3%)
Prematurity (<37 weeks)	14 (9.6%)
Genetic/Metabolic disorder	10 (6.8%)
Respiratory	8 (5.5%)
Cardiovascular	8 (5.5%)
Gastrointestinal	5 (3.4%)
Neurological	2 (1.4%)
Hematology and oncologic	1 (0.7%)
Renal	1 (0.7%)
≥ 2 chronic medical conditions	9 (6%)

Standard error- SE.

^a^ Median income: $89999.50. Income was originally recorded as income categories (ranges), but we recoded each option to the category mid-points.

^b^ Level of oxygen support are: 1) No support; (2) Standard oxygen therapy (low-flow nasal cannula and bag mask/simple face mask); (3) High-flow nasal cannula; (4) Non-invasive ventilation (continuous positive airway pressure (CPAP) or biphasic positive airway pressure (BiPAP)); and (5) Invasive mechanical ventilation (conventional positive pressure ventilation or high frequency oscillatory ventilation (HFOV) via an endotracheal tube) +/- Extracorporeal Membrane Oxygenation (ECMO) (5).

### Dependent variables

Caregiver psychological burden is operationalized by *stress* and *anxiety. Stress* is measured with the PSS, which was developed by Miles [20]. It uses a set of 26 items to gauge caregiver stress related to the hospitalization of a child on a scale of 1 (not at all stressful) to 5 (extremely stressful). We created two indexes based on the original subscales suggested by Miles—the *sight and sound* and *child looks* *and* *behavior indexes.* The *sight and sound index* (alpha reliability = 0.72) is comprised of the first 5 PSS items (**Table 2**); it excludes Item 4 due to fewer data points. It measures the extent to which the hospital environment represents a source of caregiver stress. The *child looks and behavior* index (alpha reliability = 0.83) comprises Items 6–19, although several items were similarly not used because of fewer data points. *Anxiety* is measured with the short-form STAIAD instrument, which entails 20 items used to measure anxiety on a scale of 1 (not at all) to 4 (very much so). From these items, we created 3 summative indexes—positive outlook, negative outlook, and composite. The *positive outlook* index (alpha reliability = 0.86) is made up of 7 items that represent desirable traits, such as, *I feel calm* and *I feel steady*, while the *negative outlook* index (alpha reliability = 0.89) is made up of the remaining 13 items representing traits that are not desirable, such as, *I am worried* and *I feel nervous*. The *composite index* (alpha reliability = 0.92) is created from all 20 items (**Table 3**). Before creating this index, positive outlook items were reverse-coded: 1 = 4; 2 = 3; 3 = 2; and 4 = 1. A higher score on the positive outlook index suggests less stress, while a higher score on the negative outlook and composite indexes suggests more stress.

**Table 2:** Parental stress as measured with the Parental Stress Scale among caregivers of children ≤2 years of age hospitalized with LRTI RSV infection 2019-2022 (N = 146).

			Percent Reporting		
**Variable**	**Mean** ^**b**^	**Std. error**	**Not at all** **stressful**	**Little or mod.** **stressful**	**Very or extremely** **stressful**
***Individual Items***					
1). Presence of monitors and equipment	2.18	0.10	36.99	48.63	14.38
2). Constant noise of monitors & equipment	2.39	0.11	30.00	49.29	20.71
3). The sudden noise of monitor alarm	3.18	0.11	14.08	47.18	38.74
4). The other sick babies in the room	3.00	0.23	30.61	24.49	44.90
5). The large number of people working in the unit	1.55	0.08	70.45	21.97	7.58
6). Tubes and equipment on or near my child	3.57	0.10	8.28	37.34	54.48
7). Bruises, cuts or incisions on my child	2.98	0.15	19.78	40.66	39.56
8). The unusual color of my child	3.44	0.13	10.09	43.12	46.79
9). My child’s unusual or abnormal breathing pattern	4.32	0.07	2.08	12.50	85.42
10). The small size of my child	2.78	0.22	32.00	30.00	38.00
11). The wrinkled appearance of my child	2.95	0.23	19.51	43.90	36.59
12). Having a machine (respirator) breathe for my child	4.08	0.33	0	30.77	69.23
13). Seeing needles and tubes put in my child	3.81	0.12	8.41	24.30	67.29
14). My child being fed by an intravenous line or tube	2.77	0.25	25.81	45.16	29.03
15). When my child seemed to be in pain	4.22	0.08	29.03	19.57	78.99
16). When my child looked sad	3.93	0.09	0.70	32.87	66.43
17). The limp and weak appearance of my child	3.95	0.10	3.85	23.08	73.08
18). Jerky or restless movements of my child	3.46	0.11	7.63	40.68	51.69
19). My baby not being able to cry like other children	3.87	0.11	5.26	32.46	62.28
20). Being separated from my child	4.00	0.13	7.61	20.65	71.74
21). Not feeding my child myself	3.78	0.19	4.88	39.02	56.10
22). Not being able to care for my child myself	2.70	0.21	25.00	47.73	27.27
23). Not being able to hold my child when I want	3.76	0.21	5.41	27.03	67.57
24). Feeling helpless and unable to protect my child from pain and painful procedures	4.30	0.09	3.52	15.49	80.99
25). Feeling helpless about how to help my child during this time	4.38	0.08	1.40	13.29	86.57
26). Not having time alone with my child	3.22	0.32	22.73	27.27	50.00
**Indexes**					
Sight and sound (Min. = 4; Max. = 18)—original subscale	9.34	0.29	–	–	–
Child looks & behavior (Min. = 7; Max. = 25)—original subscale	20.15	0.36	–	–	–

^a^Discrepancy in N across the different items is due to item nonresponse (i.e., respondents failing to answer some questions, which may be intentional or unintentional)

^b^Means based on scale ranging from 1 (not at all stressful) to 5 (extremely stressful).

**Table 3:** Parental anxiety as measured with the State-Trait Anxiety Inventory for Adults (STAIAD) among caregivers of children ≤2 years of age hospitalized with RSV infection, 2019-2022.

	During Admission (N=146)		Post-Discharge (N=109)		Difference in Mean (During Adm.-Post-Adm.)	t	Sig. Prob.
STAID Item	Mean	Std. Error	Mean	Std. Error			
***Positive outlook*** ^***a***^							
I feel calm	2.68	0.10	2.95	0.08	-0.27	-2.96	0.004
I feel at ease	2.18	0.11	2.65	0.10	-0.47	-3.88	0.000
I am relaxed	2.16	0.11	2.62	0.10	-0.46	-4.44	0.000
I feel Steady	3.34	0.08	3.19	0.08	0.15	1.61	0.110
I feel satisfied with myself	2.73	0.11	2.94	0.10	-0.21	-1.77	0.080
I feel secure	3.31	0.09	3.06	0.09	0.25	2.61	0.010
I am a steady person	2.59	0.11	2.76	0.10	-0.17	-1.430	0.156
***Negative outlook*** ^***a***^							
I am worried	2.85	0.11	2.17	0.09	0.69	6.53	0.000
Worry: things that don’t matter	2.71	0.12	1.81	0.09	0.91	6.71	0.000
I feel nervous	2.49	0.11	1.78	0.09	0.71	5.91	0.000
I feel nervous and restless	2.49	0.12	1.79	0.09	0.70	5.79	0.000
I am tense	2.37	0.12	1.94	0.09	0.44	3.69	0.000
I get in state of tension and turmoil	2.18	0.12	1.99	0.10	0.19	1.83	0.070
Worry over misfortunes	2.10	0.12	2.31	0.11	-0.21	-1.41	0.162
I feel frightened	1.94	0.11	1.44	0.08	0.50	4.18	0.000
I feel jittery	1.72	0.11	1.44	0.08	0.28	2.43	0.017
I feel inadequate	1.68	0.10	1.58	0.09	0.10	1.10	0.276
Wish I could be happy as others	1.53	0.09	1.55	0.08	-0.02	-0.18	0.855
I lack self-confidence	1.48	0.08	1.47	0.08	0.01	0.13	0.900
I feel like a failure	1.32	0.07	1.35	0.07	-0.03	-0.36	0.734
**Indexes**							
Positive Outlook STAID Items index	18.68	0.45	20.15	0.51	-1.48	-2.15	0.032
Negative Outlook STAID Items index	26.91	0.76	22.41	0.78	4.50	4.08	0.000
Composite anxiety index	42.72	1.14	37.14	1.23	5.58	3.30	0.001

^a^Means of individual positive outlook and negative outlook items based on scale ranging from 1 (not at all) to 4 (very much so).

^b^Discrepancy in N across the different items is due to item nonresponse (i.e., respondents failing to answer some questions, which may be intentional or inadvertent)

### Independent variables

The main independent variables include sociodemographic characteristics, measures of illness severity, and pre-existing chronic medical conditions (CMC). The sociodemographic characteristics are caregivers’ race, ethnicity, gender, age, income, and health insurance status. *Ethnicity* was measured by whether a caregiver identifies as Hispanic or not. This approach follows the U.S. Office of Management and Budget’s (OMB) standards on ethnicity and race issued in 1997. *Race* was measured by whether a caregiver identifies as White or not. The original survey captured the full list of U.S. race groups recommended by the OMB, but we collapsed the resulting responses into White and non-White because few respondents identified as non-White. *Gender* was measured by whether a caregiver identifies as female or male. *Age* in years was caregivers reported age as of last birthday. *Household income* was caregivers reported total combined income of all household members for the prior year before taxes and deductions. *Health insurance status* was measured by whether the patient is covered by private insurance, Medicaid, or none (self-pay). Finally, *out-of-pocket cost* was measured by whether or not the caregiver reported incurring out-of-pocket non-hospital expenses during the hospitalization, such as purchasing non-prescription medications for their child or hiring a babysitter.

Hospital length of stay (LOS) and the highest level of oxygen/respiratory support a child needed during hospitalization were used to measure illness severity. *Hospital LOS* was the number of days a child was hospitalized with RSV LRTI. *Level of oxygen/respiratory* support is measured with a 5-category item gauging level of oxygen support a hospitalized child needed: 1) None; 2) Standard oxygen therapy (low-flow nasal cannula and bag mask/simple face mask); 3) High-flow nasal cannula; 4) Non-invasive ventilation (continuous positive airway pressure (CPAP) or bilevel positive airway pressure (BiPAP)); and 5) Invasive mechanical ventilation (conventional positive pressure ventilation or high frequency oscillatory ventilation [HFOV] via an endotracheal tube and/or Extracorporeal Membrane Oxygenation (ECMO) [25]. Pre-existing CMC is measured by whether or not the hospitalized child had one or more CMCs (yes = 1; no = 0). This information was abstracted from patients’ health records.

### Data analysis

We analyzed the data using both descriptive statistics and multivariate regression analysis. Paired t-tests were also used to evaluate differences in caregiver reported levels of anxiety during hospitalization and post-discharge. Multivariate regression analyses were used to examine how socio-demographic and clinical characteristics are related to caregiver PSS and STAIAD indexes. Because the PSS-related variables are one-time measures, we applied ordinary least squares regression. The equivalent of fixed effects regression is applied to the models involving the STAIAD indexes, given that these were measured at two different time-points. We refined the regression models further by considering potential interactions between the independent variables. Missing data were handled through listwise deletion, which is appropriate when missingness is random.

## Results

### Characteristics of hospitalized children and caregivers

A total of 146 caregivers completed the initial in-hospital survey, with 109 completing it at approximately 2 weeks after discharge. A summary of the demographics of caregivers and the hospitalized children are provided in **Table 1**. Among hospitalized children with RSV LRTI, 79% of caregivers were female and 82% White. Caregivers’ median age was 30 years, a mean household income of $89,417, and the overwhelming majority had private insurance (89%). Among children in the study, the median age was 6.5 months, the majority were male (55%), and 23% had at least one CMC. The median hospital LOS was 2.3 days, during which 32% of children were admitted to the ICU and 91% received supplemental oxygen/respiratory support.

### Caregiver stress and anxiety

There were substantial levels of stress and anxiety—scores exceeding the midpoint of the scale— among the caregivers (**Table 2**). Over 50% of the parents rated more than half of the PSS items used to gauge caregiver stress (15/26) as *very or extremely stressful (score of ≥4).* Over 80% rated three specific items as extremely stressful: 87% felt helpless about *how to help their child*, 85% were concerned about their child’s *unusual breathing*, and 81% felt unable to *protect their child from pain and painful procedures*. The PSS indexes also showed high level of caregiver stress (**Table 2**). The mean scores of indexes based on two of the original subscales were: 9.3 for the sight and sound index (range = 4–18); and 20.2 for the child looks and behavior index (range = 7–25).

Caregiver anxiety was also substantially elevated during hospitalization (**Table 3**). The average score for 9 of the 20 items exceeded 2.5 on a 4-point scale, while another 4 items recorded average scores that exceeded the half-way point of the scale. After discharge, there were significant differences in 11 out of 20 items that measured caregiver anxiety levels at 2 weeks. Caregivers’ average scores on 4 out of 7 items characterized as “positive outlook”, which is indicative of less or the absence of anxiety, significantly improved at approximately 2 weeks after discharge (*p < 0.05*). Similarly, the average scores on 7 out of 13 other items characterized as “negative outlook” significantly decreased between the two periods (*p < 0.05*). The negative, positive, and composite STAIAD indexes consistently showed significant changes between the in-hospital and post-discharge periods (**Table 3**).

### Multivariate regression models

Initial multivariate regression models show significant relationships between several socio-demographic variables and caregiver psychological burden (**Table 4**). Higher-income caregivers were less likely to have high stress (**Model 2**). Hispanic caregivers were less likely to score high on the positive outlook STAIAD index (**Model 3**), which means they reported higher anxiety levels, and more likely to score higher on the composite STAIAD index (**Model 5**), also indicating relative higher anxiety. Older caregivers were less likely to report higher levels of anxiety (**Model 4**). Among clinical factors included in the model, only certain oxygen/respiratory support types were significantly related to one of the stress measures. Caregivers of hospitalized children who received three kinds of oxygen/respiratory support—high-flow nasal cannula, non-invasive ventilation, and invasive ventilation + /-ECMO—experienced more stress than caregivers of children not receiving such support (**Model 1**).

**Table 4:** Multivariate linear regression of caregiver stress and anxiety indexes on socio-demographic factors, RSV illness severity, and child’s pre-existing chronic health conditions with no statistical interactions included^a^.

	Caregiver Stress Indexes (PSS Items)		Anxiety Indexes (STAIAD Items)		
	Model 1: Sight and Sound	Model 2: Child Looks and Behavior	Model 3: Positive Outlook	Model 4: Negative Outlook	Model 5: Composite
	b (S.E.)	b (S.E.)	b (S.E.)	b (S.E.)	b (S.E.)
**Caregiver socio-demographic characteristics**					
Ethnicity (Hispanic vs. non-Hispanic)	0.03 (0.08)	0.08 (0.04)	-0.18 (0.07)^*^	0.14 (0.08)	0.18 (3.13)^*^
Race (White vs. non-White)	0.01 (0.11)	0.00 (0.07)	-0.00 (0.08)	-0.01 (0.08)	0.01 (3.30)
Gender of respondent (Female vs. Male)	0.12 (0.09)	0.05 (0.06)	-0.08 (0.06)	0.06 (0.07)	0.06 (0.07)
Age of respondent, logged	-0.28 (0.19)	-0.12 (0.09)	-0.09 (0.14)	-0.36 (0.18)^*^	-0.21 (0.16)
Annual household income, logged	-0.06 (0.07)	-0.06 (0.03)^*^	0.08 (0.05)	-0.03 (0.05)	-0.04 (0.05)
Health insurance status: Medicaid (ref.=private insurance)	-0.20 (0.14)	0.08 (0.05)	-0.15 (0.13)	0.10 (0.12)	0.09 (0.12)
Health insurance status: self-pay (ref.=private insurance)	-0.14 (0.16)	-0.07 (0.07)	0.05 (0.12)	-0.10 (0.21)	-0.08 (0.16)
Hosp. out of pocket expenses (yes vs. no)	0.14 (0.08)	0.07 (0.04)	-0.06 (0.05)	0.07 (0.06)	0.07 (0.06)
**Child’s illness severity & pre-existing chronic condition**					
Length of hosp. stay in days, logged	-0.06 (0.06)	0.03 (0.03)	-0.07 (0.05)	0.07 (0.05)	0.07 (0.05)
Oxygen support: Std. oxygen therapy (ref.=none)	0.17 (0.13)	0.06 (0.07)	-0.06 (0.10)	0.03 (0.08)	0.04 (0.08)
Oxygen support: High-flow nasal cannula (ref.=none)	0.31 (0.14)^*^	0.13 (0.08)	-0.06 (0.11)	-0.02 (0.10)	0.01 (0.10)
Oxygen support: Non-invasive ventilation (ref.=none)	0.43 (0.15)^**^	0.06 (0.09)	-0.09 (0.12)	-0.02 (0.13)	0.02 (0.12)
Oxygen support: Invasive mechanical ventilation +/-ECMO (ref.=none)	0.51 (0.21)^*^	0.08 (0.10)	0.14 (0.15)	-0.11 (0.16)	-0.13 (0.16)
Chronic health condition (yes vs. no)	0.09 (0.08)	-0.02 (0.05)	-0.03 (0.07)	-0.01 (0.08)	-0.0 (0.07)
**Model Statistics**					
Intercept	3.33	3.91	2.40	4.60	4.71
N	128	117	139	131	128
R-squared	0.17	0.18	0.19	0.15	0.16

Standard errors are reported in parentheses. They are based on a robust estimation method.

^a^ The models presented here are not intended as predictive models. They simply examine how several pre-selected variables are related to stress and anxiety.

* *p* < 0.05, ^**^ *p* < 0.01, ^***^ *p* < 0.001.

**Fig 1:**
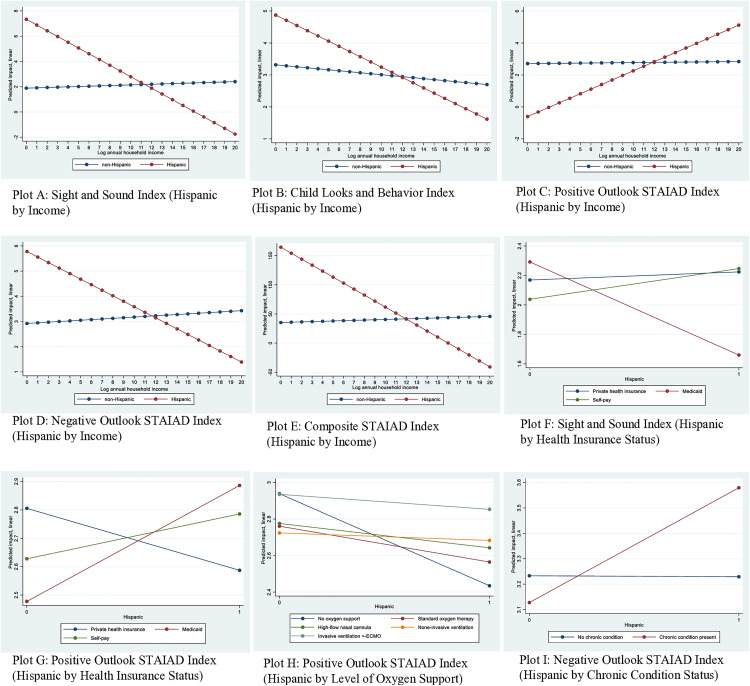
Predicted impacts of ethnicity on caregiver stress and anxiety by income, health insurance, and health. **Note**: Regression coefficients and standard errors for interactions associated with these plots are reported in Table 5.

**Fig 2:**
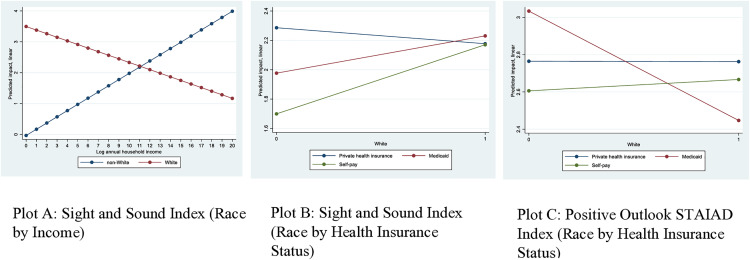
Predicted impacts of race on caregiver stress and anxiety by income and health insurance status. Note: The beta coefficients and standard errors for interactions associated with these plots are reported in Table 5.

The regression models with interaction terms, which fit the data better, revealed that some of the independent variables—ethnicity, race, household income, insurance status, level of oxygen/respiratory support, and pre-existing CMC—interact with each other to influence caregiver stress and anxiety (**Table 5, Models 1–5**). Plots of predicted marginal effects related to these interactions are reported in Figs 1–**2**. The relationships between ethnicity and caregiver stress and anxiety varied by income (**Table 5, Models 1–5**). As income increased, caregiver stress (indicated by the *sight and sound* and *child looks and behavior* indexes) decreased more rapidly among Hispanic than non-Hispanic caregivers (**Fig 1, Plots A & B**); notice that the red curve (line), which represents Hispanics, has a sharper decline than the deep blue curve (line), which represents non-Hispanics and is largely flat. Income was not significantly associated with a change in stress related to the *sight and sound* index among non-Hispanic caregivers (**Fig 1, Plot A**); notice the deep blue line remaining nearly flat (not rising or increasing) as income increased. This suggests that for non-Hispanic caregivers, income played no significantly role in shaping stress levels as represented by the *sight and sound* index. In relation to anxiety, being a Hispanic caregiver was associated with higher scores on the positive outlook STAIAD index (notice the upward trajectory of red curve in **Fig 1, Plot C**) and lower scores on the negative outlook and composite STAIAD index as income increased (notice the downward slope of the red curves in **Fig 1, Plots D & E**) as income increased. This suggests that for Hispanic caregivers, higher income makes them more likely to score higher on the STAIAD items constituting the positive outlook index, such as *I feel calm*, *I feel at ease*, *I feel steady*, and *I am relaxed*, and lower on the items constituting the negative outlook index, such as *I am worried*, *I feel nervous*, *I feel jittery*, and *I get in the state of tension and turmoil.* This was not the case with non-Hispanic caregivers. Ethnicity also interacted with health insurance status to influence both stress and anxiety. Being a Hispanic caregiver with Medicaid was negatively associated with stress (**Fig 1, Plot F,** notice the sharply declining red curv**e,** which represent Hispanics) and positively associated with the positive outlook STAIAD index (i.e., less anxiety) (**Fig 1, Plot G,** notice the sharp upward trajectory of the red curve). These results suggest that for Hispanics, having Medicaid (public health insurance) reduces stress and anxiety related to the hospitalization of a child with RSV infection. Being a Hispanic caregiver with a child receiving no oxygen/respiratory support was associated with lower scores on the positive outlook STAIAD index (**Fig 1, Plot H,** notice the sharp decline of the blue curve). The influence of a CMC on caregiver stress also differed by ethnicity. Hispanic caregivers of children with a CMC reported high stress (i.e., scored higher on the negative outlook STAIAD index) (**Fig 1, Plot I,** notice the sharp upward trajectory of the red curve). The relatively small proportion of Hispanic caregivers in the study, in some ways, points to the statistical strength of the influence of being a Hispanic caregiver on stress and anxiety. We point out, however, that based on the demographic profile of Utah, Hispanic caregivers were not underrepresented in the study.

**Table 5:** Multivariate linear regression of caregiver stress and anxiety indexes on socio-demographic factors, RSV illness severity and pre-existing chronic health conditions with statistical interactions included^a^.

	Caregiver Stress Indexes (PSS Items)		Anxiety Indexes (STAIAD Items)		
	Model 1: Sight and Sounds	Model 2: Child Looks and Behavior	Model 3: Positive Outlook	Model 4: Negative Outlook	Model 5: Composite
	b (S.E.)	b (S.E.)	b (S.E.)	b (S.E.)	b (S.E.)
**Caregiver Socio-demographic Characteristics**					
Ethnicity (Hispanic vs. non-Hispanic)	5.48 (1.30)^***^	1.56 (0.65)^*^	-3.70 (1.22)^**^	2.725(1.09)^*^	2.42 (1.07)^*^
Race (White vs. non-White)	3.48 (1.55)^*^	0.01 (0.07)	-0.00 (0.08)	0.00 (0.07)	0.02 (0.08)
Gender of respondent (Female vs. Male)	0.14 (0.09)	0.05 (0.06)	-0.08 (0.06)	0.06 (0.07)	0.05 (0.07)
Age of respondent, logged	-0.34 (0.19)	-0.16 (0.09)	0.02 (0.14)	-0.41 (0.18)^*^	-0.26 (0.17)
Annual Household income, logged	0.29 (0.14)^*^	-0.03 (0.03)	0.01 (0.05)	0.03 (0.06)	0.00 (0.06)
Health insurance status: Medicaid (ref.=private insurance)	-0.18 (0.25)	0.10 (0.06)	0.15 (0.12)	0.18 (0.16)	0.13 (0.12)
Health insurance status: self-pay (ref.=private insurance)	-0.62 (0.17)^***^	-0.06 (0.06)	-0.23 (0.29)	0.17 (0.13)	-0.08 (0.14)
Hosp. out of pocket expenses (yes vs. no)	0.15 (0.08)^*^	0.06 (0.04)	-0.03 (0.05)	0.18 (0.06)	0.06 (0.06)
**Child’s illness severity & pre-existing chronic condition**					
Length of hosp. stay in days, logged	-0.05 (0.06)	0.04 (0.03)	-0.07 (0.04)	0.09 (0.05)	0.08 (0.05)
Oxygen support: Std. oxygen therapy (ref.=none)	0.10 (0.14)	0.07 (0.07)	-0.18 (0.09)	0.01 (0.09)	0.06 (0.08)
Oxygen support: High-flow nasal cannula (ref.=none)	0.19 (0.16)	0.13 (0.08)	-0.16 (0.11)	-0.06 (0.11)	0.03 (0.11)
Oxygen support: Non-invasive ventilation (ref.=none)	0.27 (0.17)	0.05 (0.09)	-0.21 (0.11)	-0.09 (0.12)	0.01 (0.12)
Oxygen support: Invasive mechanical ventilation +/-ECMO (ref.=none)	0.38 (0.23)	0.07 (0.10)	-0.00 (0.14)	-0.20 (0.17)	-0.13 (0.16)
Chronic health condition (yes vs. no)	0.02 (0.08)	-0.02 (0.05)	-0.06 (0.08)	-0.11 (0.09)	
**Statistical Interactions**					
Ethnicity *Annual household income	-0.48 (0.12)^***^	-0.13 (0.06)^*^	0.28 (0.11)^*^	-0.24 (0.10)^*^	-0.20 ^*^
Ethnicity*Medicaid	-0.69 (0.28)^*^	NSS	0.63 (0.17)^***^	NSS	NSS
Ethnicity*Self-pay	0.15 (0.13)	NSS	0.38 (0.22)	NSS	NSS
Ethnicity* Std. oxygen therapy	NSS	NSS	0.31 (0.19)	NSS	NSS
Ethnicity* High-flow nasal cannula	NSS	NSS	0.37 (0.22)	NSS	NSS
Ethnicity* Non-invasive ventilation	NSS	NSS	0.46 (0.19)^*^	NSS	NSS
Ethnicity* Invasive mechanical ventilation +/-ECMO	NSS	NSS	0.42 (0.19)^*^	NSS	NSS
Ethnicity* Chronic health condition	NSS	NSS	NSS	0.46 (0.16)^**^	NSS
Race*Annual household income	-0.32 (0.14)^*^	NSS	NSS	NSS	NSS
Race* Medicaid	0.36 (0.26)	NSS	-0.59 (0.15)^***^	NSS	NSS
Race* Self-pay	0.58 (0.17)^***^	NSS	0.06 (0.28)	NSS	NSS
**Model Statistics**					
Intercept	-0.21	3.66	2.99	4.21	4.35
N	128	117	139	131	128
R-squared	0.32	0.20	0.32	0.21	0.18

Standard errors are reported in parentheses. They are based on a robust estimation method. ^*^ *p* < 0.05, ^**^ *p* < 0.01, ^***^ *p* < 0.001. **NSS**: These statistical interactions are excluded from the models reported because they are not statistically significant.

^a^ The models presented here are not intended as predictive models. They simply examine how several pre-selected variables are related to stress and anxiety.

The relationship between race and psychological burden also varied by income and health insurance status. Predicted margins showed that at lower levels of income, being White was associated with more stress than being non-White, but this gradually reversed as income increased (**Fig 2, Plot A,** notice that *White* starts with relatively higher *sight and sound* stress level, but this decreases rapidly as income rises, and notice the reverse for caregivers identifying as *non-White*). White caregivers who self-paid or had Medicaid reported higher levels of stress than non-Whites, while among those with private insurance, there appeared to be no significant difference between Whites and non-Whites (**Fig 2, Plot B**). The pattern remained the same for anxiety as White caregivers with Medicaid scored lower on the positive outlook STAIAD index compared with non-White caregivers (**Fig 2, Plot C**).

## Discussion and conclusion

This study provides contemporary information on caregiver psychological burdens associated with RSV-related hospitalization of children ≤2 years of age. Our findings show that RSV LRTI-related hospitalization of children is a substantial source of high levels of stress and anxiety for caregivers. Anxiety level, although less than at admission, was still reported at 2 weeks after discharge among caregivers. These findings are similar to earlier studies by Leidy et al [1] and Pokrzywinski et al. [8] of infants and children with a history of prematurity and by Mitchell et al. [26] for children <1 year-of-age with RSV hospitalization. Similar to our findings, other studies report persistence of caregiver stress and anxiety at 1 month [8] and 2 months [1] post-discharge, suggesting that the RSV hospitalization of a child is a major source of prolonged stress and anxiety for caregivers.

Secondly, several demographic and social factors in our study were associated with levels of caregiver stress and anxiety. Our results show that caregiver psychological burden is related to ethnicity (Hispanic versus non-Hispanic), race, and household income. However, the findings indicate that higher levels of stress and anxiety among Hispanics and racial minority caregivers are largely the functions of household income and health insurance status. Stress and anxiety levels among Hispanic caregivers steeply diminish as their incomes rise, while differences in stress and anxiety levels between White and non-White caregivers are apparent at the extremities of the income gradient. Being a Hispanic caregiver insured by Medicaid is associated with lower stress and anxiety, while being a White caregiver who self-pays or is insured by Medicaid is associated with more stress and anxiety. Other socio-demographic factors that influence caregiver stress and anxiety to some degree are out-of-pocket non-hospital expenses (positively) and age (negatively). Having a hospitalized child with a chronic medical condition is associated with high caregiver anxiety, but only among Hispanic caregivers.

Our findings are in line with prior studies indicating that ethnic and racial minorities, along with lower-income groups, are more likely to experience and suffer from negative health conditions and outcomes [27–31]. They are also consistent with the stress process theoretical model. The model suggests that while a trigger event, such as the hospitalization of a child, can result in stress, this consequence often differs by the social context of the affected parties. It hypothesizes that trigger events in and of themselves do not directly cause stress; they, instead, generate role strains or worsen pre-existing ones associated with affected parties’ social contexts and statuses, which in turn leads to stress [12,32]. In this study, we can conclude that the hospitalization event likely exacerbated pre-existing social strains related to being economically disadvantaged (lower-income status) and coming from an ethnic and/or racial minority background. While all the caregivers in the study experienced the same trigger event, the resulting stress and anxiety varied across the different groups.

Based on the findings of this study, we recommend heightening awareness of immunization strategies currently recommended by the U.S. Centers for Disease Control—such as nirsevimab, clesrovimab, and the maternal RSV vaccine that reduce medically attended RSV- LRTIs and RSV hospitalizations in young children [33]. Our findings further support expanded and targeted efforts to reach racial and ethnic minorities. These populations are often missed in public health campaigns, and this study suggests they suffer the most psychological burdens related to a child’s hospitalization with RSV LRTI. Given the stress and anxiety associated with RSV LRTI hospitalization in young children, the introduction and widespread use of RSV monoprophylaxis will alleviate these psychological burdens (stress and anxiety) experienced by caregivers.

There are some limitations to this study. The study might have over-sampled caregivers that were experiencing lower levels of stress; those overwhelmed by the hospitalization or other factors might have declined participation. However, review of the data on certain factors did not show significant differences between participating and non-participating respondents. Additionally, the impact and unique stressors associated with the COVID-19 pandemic were not directly examined in this study. The higher levels of stress and anxiety experienced by caregivers from ethnic and racial minorities, or those with lower household income, may have been exacerbated by the pandemic [31]. We evaluated the potential influence of this situation on our results, finding it to be inconsequential. Given the limitations identified here and the fact that the study is not based on a probability sample, the findings may not be generalizable across all caregivers of children hospitalized with RSV. Finally, hospitalization of a child for any cause, not just RSV, can lead to significant stress and anxiety among caregivers. Nevertheless, RSV is a major cause of hospitalization among generally healthy infants, making our findings important for health care providers and policymakers.

In conclusion, our study suggests that caregivers may experience significant stress and anxiety as a result of RSV-related hospitalization of their children. These psychological burdens appear to vary by socio-demographic factors such as ethnicity, race, income, and health insurance status, and by the clinical condition of the hospitalized child. The heightened levels of stress and anxiety observed in caregivers, especially within minority groups, underscore the importance of addressing equitable access to RSV immunoprophylaxis for all at-risk and healthy infants and children.
